# High Thermoelectric Power Factor of High‐Mobility 2D Electron Gas

**DOI:** 10.1002/advs.201700696

**Published:** 2017-11-24

**Authors:** Hiromichi Ohta, Sung Wng Kim, Shota Kaneki, Atsushi Yamamoto, Tamotsu Hashizume

**Affiliations:** ^1^ Research Institute for Electronic Science Hokkaido University N20W10, Kita Sapporo 001‐0020 Japan; ^2^ Graduate School of Information Science and Technology Hokkaido University N14W9, Kita Sapporo 060‐0814 Japan; ^3^ Department of Energy Science Sungkyunkwan University (SKKU) Suwon 16419 Republic of Korea; ^4^ Research Institute for Energy Conservation National Institute of Advanced Industrial Science and Technology (AIST) Umezono 1‐1‐1 Tsukuba 305‐8568 Japan; ^5^ Research Center for Integrated Quantum Electronics Hokkaido University N13W8, Kita Sapporo 060‐0814 Japan

**Keywords:** 2D electron gas, AlGaN/GaN‐MOS‐HEMT, thermoelectric power factor

## Abstract

Thermoelectric conversion is an energy harvesting technology that directly converts waste heat from various sources into electricity by the Seebeck effect of thermoelectric materials with a large thermopower (*S*), high electrical conductivity (σ), and low thermal conductivity (κ). State‐of‐the‐art nanostructuring techniques that significantly reduce κ have realized high‐performance thermoelectric materials with a figure of merit (*ZT* = *S* ^2^∙σ∙*T*∙κ^−1^) between 1.5 and 2. Although the power factor (PF = *S* ^2^∙σ) must also be enhanced to further improve *ZT*, the maximum PF remains near 1.5–4 mW m^−1^ K^−2^ due to the well‐known trade‐off relationship between *S* and σ. At a maximized PF, σ is much lower than the ideal value since impurity doping suppresses the carrier mobility. A metal‐oxide‐semiconductor high electron mobility transistor (MOS‐HEMT) structure on an AlGaN/GaN heterostructure is prepared. Applying a gate electric field to the MOS‐HEMT simultaneously modulates *S* and σ of the high‐mobility electron gas from −490 µV K^−1^ and ≈10^−1^ S cm^−1^ to −90 µV K^−1^ and ≈10^4^ S cm^−1^, while maintaining a high carrier mobility (≈1500 cm^2^ V^−1^ s^−1^). The maximized PF of the high‐mobility electron gas is ≈9 mW m^−1^ K^−2^, which is a two‐ to sixfold increase compared to state‐of‐the‐art practical thermoelectric materials.

Currently, more than 60% of the energy produced from fossil fuels is lost as waste heat. Consequently, thermoelectric energy conversion has attracted much attention as an energy harvesting technology since thermoelectric devices can directly convert waste heat from various sources such as electric power plants, factories, and automobiles into electricity.^1^ The energy conversion efficiency is generally evaluated using the dimensionless figure of merit, *ZT* = *S* ^2^∙σ∙*T*∙κ^−1^, where *Z* is the figure of merit, *T* is the absolute temperature, *S* is the thermopower (≡Seebeck coefficient), σ is the electrical conductivity, and κ is the sum of the electronic (κ_ele_) and lattice thermal conductivities (κ_lat_) of a thermoelectric material. To date, state‐of‐the‐art nanostructuring techniques, which can reduce κ_lat_ significantly through phonon scattering by nanosized structural defects,^2^ have realized high‐performance thermoelectric materials showing a large *ZT* of 1.5–2. However, κ_lat_ reduction techniques generally deteriorate σ due to the significant decrease in carrier mobility (μ), leading to a moderate net benefit in the maximized *ZT*, which is determined by the ratio of μ/κ_lat_ (quality factor).^3^

On the other hand, the product *S* ^2^∙σ, which is called the power factor (PF), is also used to evaluate a thermoelectric material. Although a low κ is strongly required when the material is placed in a thermally isolated atmosphere such as space (in a vacuum), a high PF is more critical than a low κ when the surrounding atmosphere heats and cools the material. In fact, Yamamoto et al. experimentally demonstrated that thermoelectric devices with large κ metals composed of Constantan (N‐leg) and Chromel (P‐leg) sheets efficiently generate electric power in a butane gas flame.^4^ In this case, heat transfer is governed by convection heat flow, which cancels the conduction in the material. Thus, a high PF is necessary for efficient power generation rather than low κ when a material is not in a thermally isolated atmosphere.

The PF must be optimized due to well‐known trade‐off relationship between *S* and σ in terms of the volume carrier concentration (*n*_v_); as *n*_v_ increases, σ increases, whereas |*S*| decreases. Optimized PF values of state‐of‐the‐art bulk thermoelectric materials range between 1.5 and 4 mW m^−1^ K^−2^ at room temperature, without exception of recently reported Nb_1−_ *_x_*Ti*_x_*FeSb half‐Heusler (10.6 mW m^−1^ K^−2^).^5^ Examples include SnSe (PF ≈ 4 mW m^−1^ K^−2^),^6^ Bi_0.5_Sb_1.5_Te_3_ (PF ≈ 4 mW m^−1^ K^−2^),[[qv: 2b,c]] AgPb*_m_*SbTe_2+_ *_m_* (3.6 mW m^−1^ K^−2^),[[qv: 2a]] CsBi_4_Te_6_ (3.4 mW m^−1^ K^−2^),^7^ SiGe alloy (1.5 mW m^−1^ K^−2^),^8^ Na_0.88_CoO_2_ (3.4 mW m^−1^ K^−2^),^9^ Nb‐doped SrTiO_3_ (≈2.5 mW m^−1^ K^−2^),^10^ PbTe‐SrTe (1.4 mW m^−1^ K^−2^),^11^ and rough Si nanowire (PF ≈ 3 mW m^−1^ K^−2^).^12^ Thus, the PF enhancement is limited by difficulties realizing a simultaneous increase in *S* and σ. Additionally, there is another trade‐off relationship between the carrier mobility (μ) and *n*_v_; σ at the maximized PF is much lower than the ideal value since impurity doping significantly suppresses μ (**Figure** **1**a). In a typical semiconductor, μ decreases ≈1/10 by conventional impurity doping.^13^

**Figure 1:**
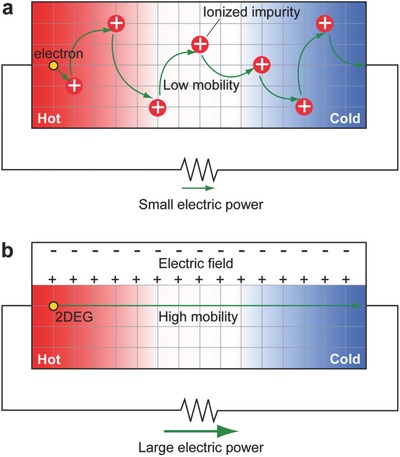
Schematic illustration of thermoelectric power generation in an n‐type semiconductor. a) Conventional impurity doped n‐type semiconductor. Carrier electron flow from the hot to the cold side due to the Seebeck effect. Low mobility of the carrier electron originates from ionized impurity scattering. b) Electric field induced high‐mobility 2DEG. Larger electric power can be obtained compared with (a).

Herein we propose that a high‐mobility 2D electron gas (2DEG) at a semiconductor heterointerface is a viable solution to overcome the bottleneck in thermoelectric trade‐off relations. In 2DEG, μ is not suppressed since the high‐mobility channel lacks an impurity (Figure 1b). Furthermore, the PF by the electric field carrier concentration modulation can be optimized by the metal‐oxide‐semiconductor (MOS) structure on such a 2DEG. In this study, we investigate the PF of a 2DEG, which is induced at an AlGaN/GaN heterointerface by the electric field thermopower modulation method.^14^ The maximized PF of the 2DEG is ≈9 mW m^−1^ K^−2^ at room temperature, which is an order magnitude greater than that of the doped GaN bulk and two‐ to sixfold greater than those of state‐of‐the‐art thermoelectric materials, while maintaining a higher σ (= 6030 S cm^−1^) than state‐of‐the‐art thermoelectric materials (σ = 1000–2500 S cm^−1^).

We fabricated an Al_2_O_3_/AlGaN/GaN metal‐oxide‐semiconductor high electron mobility transistor (MOS‐HEMT)^15^ and measured the thermoelectric properties of the 2DEG by introducing a temperature difference during a gate voltage (*V*_g_) application to modulate the Fermi energy (*E*_F_) (**Figure** **2**a). We used a commercially available Al_0.24_Ga_0.76_N (20 nm)/GaN (900 nm)/Fe‐doped GaN (300 nm) heterostructure film, which was grown on a semi‐insulating (0001) SiC substrate by metal organic chemical vapor deposition (Figure 2b). The sheet resistance (*R*_s_), Hall mobility (μ_Hall_), and sheet carrier concentration (*n*_s_) were 423 Ω sq^−1^, 1730 cm^2^ V^−1^ s^−1^, and 8.53 × 10^12^ cm^−2^, respectively, at room temperature. The gate length (*L*) and width (*W*) of the MOS‐HEMTs were 800 µm and 400 µm, respectively. Details of our MOS‐HEMT preparation are described in the Experimental Section and elsewhere.^15^

**Figure 2:**
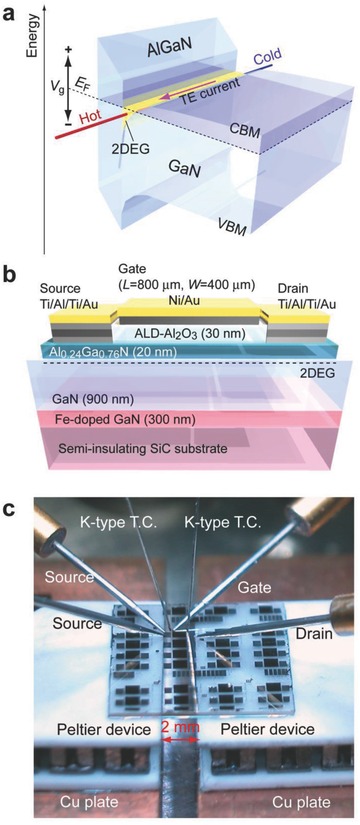
Electric field thermopower modulation measurement of AlGaN/GaN MOS‐HEMT. a) Schematic energy band diagram of 2DEG confined at an AlGaN/GaN heterointerface. CBM and VBM denote the conduction band minimum and the valence band maximum, respectively. Thermoelectric properties can be modulated by applying a gate voltage (*V*_g_). b) Schematic illustration of the AlGaN/GaN MOS‐HEMT. c) Photograph of the electric field thermopower modulation measurement of AlGaN/GaN‐MOSHEMT. MOSHEMT is placed between the gap (2 mm) of two Peltier devices. During a gate voltage application, temperature differences (Δ*T*) are introduced between both ends of the channel using the Peltier devices.

Figure 2c shows the carrier transport properties at room temperature. The transistor characteristics were measured using a semiconductor device analyzer (B1500A, Agilent). For the *S* measurements, we used two Peltier devices placed under the MOS‐HEMT with a 2 mm gap to induce a temperature difference between the source and drain electrodes (Δ*T*, 0–1 K), which was monitored via two thermocouples (K‐type, 150 µm diameter, SHINNETSU Co.) mechanically attached at both edges of the 2DEG channel. The thermo‐electromotive force (Δ*V*) and Δ*T* were simultaneously measured at room temperature. Then the slope of the Δ*V*–Δ*T* plots yielded the *S*‐values. Details of our electric field modulated *S* measurement are described elsewhere.[[qv: 14b–d]]

**Figure** **3** summarizes the carrier transport properties of the MOS‐HEMT at room temperature. Applying a gate voltage (*V*_g_) from −9 to +4 V at a constant drain voltage (*V*_d_) of +10 V dramatically modulates the drain current (*I*_d_) from 7 nA to 7 mA (≡on‐to‐off current ratio ≈10^6^) (Figure 3a). The gate leakage current (*I*_g_) is ≈300 pA when *V*_g_ is less than +2 V, while the *I*_d_^0.5^ versus *V*_g_ plot indicates that the threshold gate voltage (*V*_th_) is −7.98 V. The gate capacitance per unit area (*C*_i_) is 166.3 nF cm^−2^ (Figure 3a, inset). The output characteristic curves clearly show the pinch‐off behavior and the current saturation of *I*_d_ (Figure 3b), indicating that the characteristic of the MOS‐HEMT obeys the standard transistor theory.

**Figure 3:**
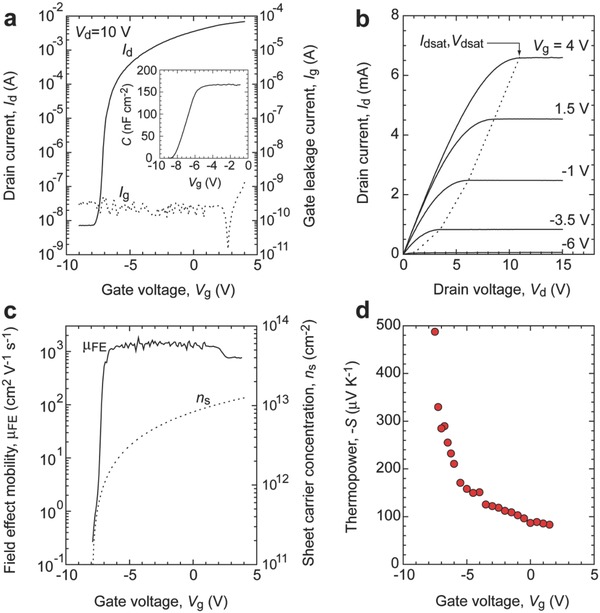
Carrier transport properties of 2DEG in AlGaN/GaN MOS‐HEMT at room temperature. a) Transfer *I*_d_–*V*_g_ characteristic at *V*_d_ = 10 V. *I*_g_–*V*_g_ characteristic is also plotted. Inset shows the *C*_i_–*V*_g_ characteristic. b) Output *I*_d_–*V*_d_ characteristics (−6 V ≤ *V*_g_ ≤ + 4 V). Pinch‐off and current saturation behaviors are clearly observed. c) Changes in the field effect mobility (μ_FE_) and the sheet carrier concentration (*n*_s_) as functions of *V*_g_. d) Change in the thermopower (*S*) as a function of *V*_g_.

The *n*_s_ value was calculated as *n*_s_ = *C*_i_·(*V*_g_−*V*_th_)·*e* ^−1^. At *V*_g_ = 0 V, its value is 8.32 × 10^12^ cm^−2^, which agrees well with that obtained from the Hall measurement (*n*_s_ = 8.53 × 10^12^ cm^−2^) (Figure 3c). In the present MOS‐HEMT, *n*_s_ can be modulated from ≈10^11^ cm^−2^ up to 1.25 × 10^13^ cm^−2^. The field effect mobility (μ_FE_) was calculated as

(1) $$\mu_{\mathrm{FE}}=\frac{g_{m}\cdot L}{C_{i}\cdot (V_{g}-\;V_{\mathrm{th}})\cdot W}$$

where *g*_m_ is the transconductance, d*I*_d_/d*V*_g_. μ_FE_ drastically increases from ≈10^−1^ to ≈10^3^ cm^2^ V^−1^ s^−1^ around *V*_g_ ≈ −7 V, and is almost saturated at ≈1500 cm^2^ V^−1^ s^−1^ when −7 V < *V*_g_ < +2 V, similar to that of the original value (1730 cm^2^ V^−1^ s^−1^) obtained from the Hall measurement (Figure 3c). These results clearly demonstrate that the 2D carrier concentration can be modulated without suppressing the mobility in the MOS‐HEMT.

Next, we measured *S* of the MOS‐HEMT as a function of *V*_g_ (Figure 3d). The observed *S* values are always negative, indicating that the channel is an n‐type semiconductor. As *V*_g_ increases, |*S*| monotonically decreases from 490 to 90 µV K^−1^. The observed *S* values reflect a bulk‐like energy derivation of the parabolic shaped density of states (DOS) of the conduction band near the Fermi energy. The observable *S* is roughly expressed as *S*_obs_ = (σ_s2DEG_·*S*_2DEG_ + σ_sBulk_·*S*_Bulk_)/(σ_s2DEG_ + σ_sBulk_), where σ_s_ is the sheet conductance of each layer. In the present case, *S*_2DEG_ dominates *S*_obs_ due to the relation of σ_s2DEG_ ≫ σ_sBulk_.

Since the energy dependence of the DOS near the conduction band bottom for GaN is parabolic, the *n* dependence of *S* for electron‐doped GaN bulk can be theoretically calculated using the following equations (2)–(4), 16

(2) $$n_{-}=4\pi {\left(\frac{2m*k_{B}T}{h^{2}}\right)}^{3/2}F_{1/2}\left(\xi \right)$$

(3) $$F_{r}\left(\xi \right)=\int_{0}^{\infty }\frac{x^{r}}{1+e^{x-\xi }}dx$$

(4) $$S=-\frac{k_{B}}{e}\left(\frac{\left(r+2)F_{r+1}(\xi \right)}{\left(r+1)F_{r}(\xi \right)}-\xi \right)$$

where m*, *k*_B_, *T*, *h*, ξ, *F_r_*, and *r* are the DOS effective mass, Boltzmann constant, absolute temperature, Planck constant, chemical potential, Fermi integral, and scattering parameter of relaxation time, respectively. We used 0.19 *m*_0_ 17 as m* of GaN, where *m*_0_ is the free electron mass. **Figure** **4** shows the calculated *S* of bulk GaN as a function of the volume carrier concentration (*n*_v_) at 300 K. For comparison, several reported *S* values of electron‐doped GaN are also plotted (Brandt,^18^ Sztein,^19^ Nagase^20^). The calculated line completely reproduces these reported values. Thus, we used this *S*–*n*_v_ relationship to calculate the PF of the 2DEG layer.

**Figure 4:**
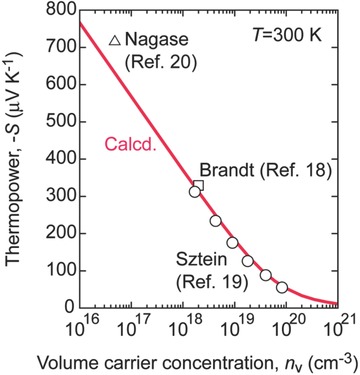
Relationship between thermopower and volume carrier concentration (logarithmic scaled) of bulk GaN. Theoretically calculated *S* of electron‐doped GaN with a parabolic‐shaped energy dependence of DOS around the conduction band bottom (*T* = 300 K). Experimentally obtained values, which are reported by Sztein et al.,^19^ Brandt et al.,^18^ and Nagase et al.,^20^ are plotted for comparison. Calculated line completely reproduces these reported values.

The PF of the AlGaN/GaN 2DEG was calculated using the observed *S*, *n*_v_ obtained from Figure 4, and the observed μ_FE_ (**Figure** **5**a). A high PF of ≈9 mW m^−1^ K^−2^ with *n*_v_ ≈ 2.5 × 10^19^ cm^−3^ is calculated in high‐mobility 2DEG at the AlGaN/GaN interface at room temperature, which is an order magnitude greater than that of doped GaN bulk^19^ and a two‐ to sixfold increase compared to those of state‐of‐the‐art practical thermoelectric materials (1.5–4 mW m^−1^ K^−2^). The carrier mobility of AlGaN‐GaN 2DEG at *n*_v_ ≈ 2.5 × 10^19^ cm^−3^ is ≈1500 cm^2^ V^−1^ s^−1^, which is an order magnitude larger than that of conventional impurity doped bulk GaN (≈125 cm^2^ V^−1^ s^−1^).

**Figure 5:**
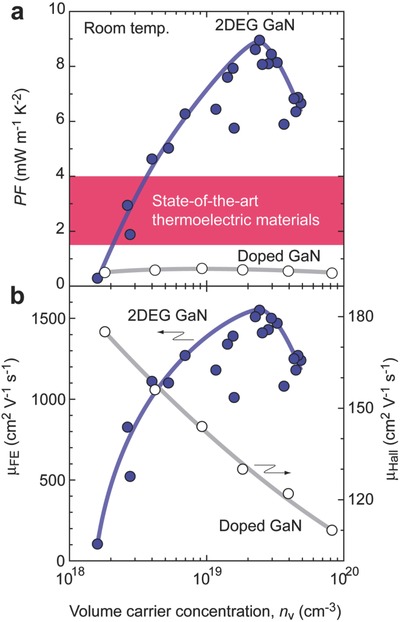
High thermoelectric power factor of high‐mobility 2DEG at AlGaN/GaN interface. Carrier concentration dependence of a) PF and b) carrier mobility (μ) for 2DEG GaN and conventional impurity doped n‐type GaN. Maximized PF of the 2DEG with *n*_v_ ≈ 2.5 × 10^19^ cm^−3^ is ≈9 mW m^−1^ K^−2^ at room temperature, which is an order magnitude greater than that of doped GaN bulk^19^ and a factor of 2–6 greater than that state‐of‐the‐art practical thermoelectric materials (1.5–4 mW m^−1^ K^−2^).

Finally, we would like to discuss the effective thickness (*t*_eff_) of 2DEG at AlGaN/GaN. **Figure** **6**a shows *n*_v_ as a function of *V*_g_. We defined *t*_eff_ as *n*_s_/*n*_v_ as shown in the inset. *t*_eff_ dramatically decreases from 20 to ≈2 nm with *V*_g_ when *V*_g_ < −6 V (subthreshold region), whereas it is almost saturated at a constant value (≈2 nm), which agrees well with the previously reported 2DEG thickness,^21^ when *V*_g_ > −6 V (Figure 6b). The thermal de Broglie wavelength (λ_D_) of GaN is ≈10 nm, which can be calculated by the following equation

(5) $$\lambda_{D}=\frac{h}{\sqrt{3\cdot m*\cdot k_{B}\cdot T}}$$

**Figure 6:**
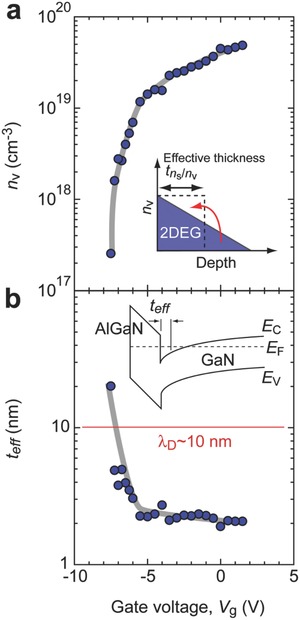
Effective thickness of 2DEG at the AlGaN/GaN interface. Changes in a) the volume carrier concentration (*n*_v_) and b) the effective thickness, which is defined as *n*_s_/*n*_v_, as a function of *V*_g_. *n*_v_ means the average volume carrier concentration of 2DEG as schematically shown in the inset of (a). Definition of the effective thickness is shown in the inset of (b).

In our result, *t*_eff_ strides over λ_D_. Thus, an enhanced *S* can be expected because it is theoretically predicted that a quantum well narrower than λ_D_ will exhibit an enhanced *S*.^22^ In the case of SrTiO_3_‐based 2DEG, a V‐shaped upturn of *S* is observed when the 2DEG thickness is narrower than ≈2 nm, clearly demonstrating the theory. However, such an *S* behavior is not observed in the present AlGaN/GaN 2DEG, indicating that any special effect of 2DEG does not contribute to the observed *S*.

In summary, we experimentally clarified that the high‐mobility 2D electron gas induced at an AlGaN/GaN heterointerface exhibits a high thermoelectric power factor PF of ≈9 mW m^−1^ K^−2^ at room temperature, which is an order magnitude greater than that of doped GaN bulk and a factor of 2–6 compared to those of state‐of‐the‐art practical thermoelectric materials (1.5–4 mW m^−1^ K^−2^). Although the present AlGaN/GaN cannot be used as the thermoelectric generator because of its narrow thickness, the present high‐mobility electron gas approach should open an avenue to further improve the thermoelectric performance of state‐of‐the‐art thermoelectric materials.

## Experimental Section

*Preparation of Al_2_O_3_/AlGaN/GaN MOS‐HEMT*: An Al_0.24_Ga_0.76_N (20 nm)/GaN (900 nm)/Fe‐doped GaN (300 nm) heterostructure film grown on a semi‐insulating (0001) SiC substrate via metal organic chemical vapor deposition was used. The sheet resistance, Hall mobility, and sheet carrier concentration were 423 Ω sq^−1^, 1730 cm^2^ V^−1^ s^−1^, and 8.53 × 10^12^ cm^−2^, respectively, at room temperature. As an ohmic electrode, a multilayer consisting of Ti/Al/Ti/Au was deposited on the AlGaN surface and subsequently annealed at 830 °C for 1 min in N_2_ ambient. A 20 nm thick SiN*_x_* film was used as a surface protection layer to mitigate damage to the AlGaN surface during ohmic annealing.^15^, ^23^ After forming the ohmic electrode, the SiN*_x_* layer was removed in a buffered HF solution. An Al_2_O_3_ layer with a nominal thickness of 30 nm was then deposited on the AlGaN surface at 300 °C using an atomic layer deposition (ALD) system. Trimethylaluminum and water vapor were introduced into an ALD reactor in alternate pulse form as the aluminum and oxygen precursors, respectively. The deposition rate was 0.11 nm cycle^−1^. Finally, a Ni/Au (20/50 nm) gate electrode was formed on the Al_2_O_3_ layer. To improve the Al_2_O_3_/AlGaN interface properties, the sample was annealed at 300 °C for 3 h in air under a reverse bias of −10 V.^24^ The gate length (*L*) and width (*W*) of the MOS‐HEMTs were 800 µm and 400 µm, respectively.

*Measurements*: The transistor characteristics of the AlGaN/GaN MOS‐HEMT were measured using a semiconductor device analyzer (B1500A, Agilent) at room temperature in air. For the *S* measurements, two Peltier devices were used, which were placed under the MOS‐HEMT, to generate a temperature difference between the source and the drain electrodes. Two thermocouples (K‐type, 150 µm diameter, SHINNETSU Co.), which were mechanically attached at both edges of the 2DEG channel, monitored the temperature difference (Δ*T*, 0–1 K). The thermo‐electromotive force (Δ*V*) and Δ*T* values were simultaneously measured at room temperature, and the slope of the Δ*V*–Δ*T* plots yielded the *S*‐values. The error of *S* should be less than 5%. Details of electric field modulated *S* measurement are described elsewhere.[[qv: 14b–d]]

## Conflict of Interest

The authors declare no conflict of interest.
